# Nutritional and Clinical Factors Affecting Weight and Fat-Free Mass Loss after Gastrectomy in Patients with Gastric Cancer

**DOI:** 10.3390/nu12071905

**Published:** 2020-06-27

**Authors:** Hee-Sook Lim, Bora Lee, In Cho, Gyu Seok Cho

**Affiliations:** 1 Department of Food and Nutrition, Yeonsung University, Anyang 14011, Korea; limhs@yeonsung.ac.kr; 2 Department of Statistics, Chung-Ang University, Seoul 06974, Korea; mintbora0125@gmail.com; 3 Department of Surgery, Soonchunhyang University Bucheon Hospital, Soonchunhyang University College of Medicine, Bucheon 14584, Korea; gschogs@schmc.ac.kr

**Keywords:** stomach neoplasm, gastrectomy, body weight loss, diet therapy, calorie intake

## Abstract

After a gastrectomy, the nutritional status of patients with gastric cancer has great effect on the treatment outcome and patients’ quality of life. We investigated the changes in body composition and nutrient intake after gastrectomy in 288 gastric cancer patients. A multiple linear regression analysis was used for each time period to verify the effects of nutritional and clinical factors on weight and fat-free mass loss rates. Gastric cancer patients who underwent a gastrectomy continued to experience weight and fat-free mass loss until three months after surgery and became stagnant at six months. The marginal mean of calorie intake per weight was 24.5, 26.8, and 29.4 kcal at one, three, and six months. The protein intake per kg lean mass was 1.14, 1.14, and 1.16 g at one, three, and six months, respectively. One month after surgery, the rate of weight loss increased significantly in females who received chemotherapy (*p* < 0.001). At one to three months postoperative, females who had undergone chemotherapy tended to significantly lose weight (*p* = 0.016). Females with a history of chemotherapy also showed a reduction in fat-free mass for one to three months (*p* = 0.036). Calorie intake was a significant factor in preventing fat-free mass weight loss at one month after surgery. Chemotherapy was an independent factor affecting the weight and fat-free mass loss rate up to six months after gastrectomy. Careful monitoring of weight and muscle mass changes following active nutritional intervention for sufficient nutrition support could be helpful for patients after gastrectomy.

## 1. Introduction

Gastric cancer is one of the most common malignancies and the third most common cause of cancer-related deaths worldwide [1]. In Korea, the 5-year relative survival rate of gastric cancer increased drastically from 42.8% in the early 1990s to 75.4% in 2011–2015 [2]. This increase is attributed to early detection and the development of treatment. In addition, quality of life and nutritional management may affect the survival rate of patients who undergo gastric cancer surgery [3].

There are several reports showing that malnutrition after gastrectomy occurs with a wide range of frequency, from 20% to 61%, concurrently with micronutrient deficiency [4,5,6]. Nutritional problems commonly occurring among patients after gastrectomy include dumping syndrome, weight loss, and anemia [7]. The main symptoms include anorexia, reflux, bloating, vomiting, and diarrhea. Moreover, problems with malnutrition may continue to be related to the patient’s quality of life [8]. Malnutrition among patients with gastric cancer is not only due to malabsorption but also due to a lack of food intake. The intake from meals is generally less than the recommended requirements, leading to weight loss and nutritional imbalances. Thus, patients experience generalized weakness, causing reduced activity [9,10]. In a study of 1418 gastric cancer patients, 21.4% of the subjects were underweight one year after the gastrectomy [11]. Another study reported that hypoproteinemia and hypoalbuminemia were found in 58% of the subjects six to nine months after gastrectomy. In addition, the body mass index (BMI) decreased significantly in 98.3% of patients who underwent gastrectomy [12]; only 10.5% of patients who underwent gastrectomy recovered to their usual weight [13]. Indicators for assessing the nutritional status of gastric cancer patients include nutritional assessment tools, biochemical tests, and nutrient intake status; however, a more objective indicator is weight loss. The majority of cancer patients are not able to achieve a positive energy balance and, in many cases, cannot maintain their initial body weight, resulting in tissue wasting and muscle degradation [14,15]. Muscle mass assessment gas has garnered important attention as a method of nutritional assessment [14]. Low muscle mass is associated with a higher incidence of postoperative complications in cancer patients and is also associated with a poor long-term prognosis for patients undergoing surgery for upper gastric cancer [14,16].

In general, patients with gastrointestinal cancer need dietary therapy to control the amount or type of food they ingest, along with mental stress therapy to manage the strain from long-term treatment and anxiety about cancer recurrence. It is well known that such limitations in daily life and related psychosocial factors can delay medical treatment and recovery [17].

Gastric cancer patients that undergo gastrectomy must maintain good nutrition before surgery, prevent serious weight loss after surgery, and eat a balanced diet for recovery. To achieve these dietary requirements, a multidisciplinary approach and personalized nutritional management are necessary. Individual nutritional education was significantly more effective than group education among patients undergoing gastrectomy due to weight gain and an increase in BMI [18]. In addition, sufficient caloric intake was deemed the most important factor for rapid recovery [19]. 

Current nutritional research on gastric cancer patients has included the relationship between nutrition and quality of life [20], the efficacy of nutritional assessment tools for predicting complications [21,22], and postoperative changes in nutritional intake [23,24]. Nevertheless, there are very few studies on the dietary factors that influence the weight loss of patients over an extended time period, for both domestic and overseas patients. We thus investigated weight loss, the most important indicator of nutritional status, and the changes in nutritional intake observed over the postoperative period. Additionally, we attempted an analysis of factors influencing weight loss.

## 2. Materials and Methods 

### 2.1. Study Subjects

The subjects were gastric cancer patients selected from the “Gastric Cancer Patients Registry” at the institution. Patients who underwent gastrectomy between March 2010 and February 2015 with nutritional management were included in the study. We retrospectively reviewed the medical records of patients with good nutritional status before surgery, who took an oral diet without additional nutritional support after surgery, and who had no postoperative history of hospitalization or surgery due to complications. The final analysis was performed on 165 males and 123 females after the exclusion of cases that did not meet the selection criteria or had missing variables (a lack of weight or caloric and protein intake documentation at more than two time points). This study was conducted with the approval of the Institutional Review Board of Soonchunhyang University Bucheon Hospital under Helsinki Research Principles (approval number: SCHBC_IRB_2012-1). 

### 2.2. Data Collection

The factors used in the analysis were gender, age, BMI, waist circumference, triceps skin fold thickness (TSF), fat mass (FM), and fat-free mass (FFM). The measurements of body composition were performed at each follow-up time using a bioelectrical impedance analyzer (Biospace In-body 720, Seoul, Korea). Applying the checked height and weight, we then calculated the body mass index (BMI, kg/m^2^). The FFM was calculated by subtracting fat mass from the body weight {FFM = body weight − (body weight × fat%)}. The TSF was measured using a skin thickness gauge at the thinnest portion of the upper arm. A tape measure was used between the ribs and pelvic iliac bones in an upright posture to obtain the waist circumference. Surgical approaches were classified into laparoscopic surgery and open surgery. 

Standardized tools are widely used to assess the nutritional status of patients with gastric cancer. In order to implement more realistic nutritional interventions, it is more important to evaluate the nutritional intake of each patient and tailor their nutritional management to reach their goals than to rely on the results of the integrated assessment. For nutritional intake, a professional nutritionist analyzed dietary intake using the CAN program (Computer Aided Nutritional analysis program ver. 4.0, The Korean Nutrition Society, Seoul, Korea) based on the contents written over the week in a dietary diary prepared by each patient [25]. Accordingly, the calorie and protein intake indicated the amount per kg lean mass of patients.

### 2.3. Statistical Analyses

The demographic characteristics and clinical factors of the subjects were summarized by the mean and standard deviation for continuous variables and the frequency and percentage (%) for categorical variables. To analyze the rate of weight loss, FFM loss, and the effects of clinical factors at one, three, and six months after surgery, a mixed-model repeated measures (MMRM) approach was applied. In this model, the postoperative time and individual clinical factors (gender, surgical approach, TNM, and adjuvant chemotherapy) were considered the main effects. The individual study subjects, including the interactive effects on each clinical factor, were random effects. The postoperative period and age were the adjustment variables. The changes in caloric and protein intake at each point were also analyzed using the same model. Subsequently, a multiple linear regression model was fitted to determine the different effects of the clinical factors on the rate of weight and FFM loss at one month, one to three months, and three to six months after surgery. All the analyses were carried out using R (version 3.6.1, The R Foundation for Statistical Computing, Vienna, Austria). The statistical significance was set at 0.05 based on a two-sided test.

## 3. Results

### 3.1. Patient Characteristics 

The average age of the 288 subjects was 56.2 ± 13.1 years, with females accounting for 42.7%. The average preoperative measurements included a BMI of 23.9 ± 3.5 kg/m^2^, a waist circumference of 85.0 ± 8.0 cm, and a tricep thickness of 15.5 ± 6.5 mm. The FFM was collected in males (51.6 ± 9.5 kg) and females (45.7 ± 8.9 kg). Laparoscopic surgery was more common than open surgery (61.1%). The most common tumor location was in the lower third of the stomach (68.8%). Early gastric cancer occurred in 62.5% of cases and advanced gastric cancer in 37.5%. The number of subjects who received chemotherapy was 71 (24.7%). (Table 1) 

### 3.2. Comparison of the Weight Loss Rate According to the Postoperative Period by Clinical Factors 

**Figure 1:**
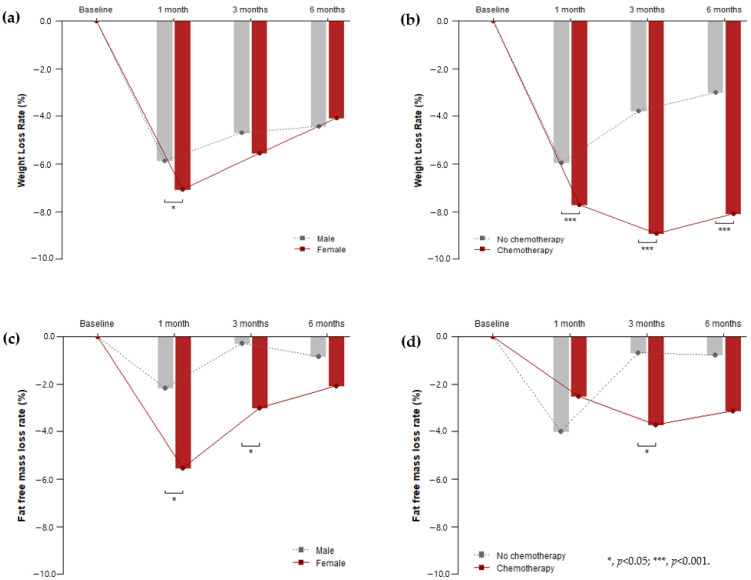
Change in weight/fat-free mass loss rate after gastrectomy according to gender and chemotherapy. (**a**) The difference in weight loss rates between males and females was statistically significant at one month after surgery (*p* = 0.012); (**b**) chemotherapy showed a significant effect on the weight loss rate over the entire postoperative period (*p* < 0.001); (**c**) the difference in the fat-free mass loss rates between males and females was statistically significant at one and three months after surgery (*p* = 0.006 and 0.033, respectively); (**d**) chemotherapy showed a significant effect on the fat-free mass loss rate at three months after surgery (*p* = 0.042).

Sex, operative approach, and cancer stage (TNM) did not have a significant effect on the rate of postoperative weight loss, unlike chemotherapy. The weight loss rate of males was 5.86% (95% CI, 6.40% to 5.33%) at one month postoperative, while females showed a larger drop at 7.07% (95% CI, 7.69 to 6.45%) during the same period. One month after surgery, the difference in weight loss rates between males and females was statistically significant (*p* = 0.012); however, the rest of the time periods showed no significant difference. By contrast, chemotherapy showed a significant effect on the weight loss rate over the entire postoperative period (*p* < 0.001). At all points, the weight loss rate of subjects who had chemotherapy was significantly higher than that of subjects who did not. The interaction between time and chemotherapy was also significant (*p* < 0.001). The pattern of change differed depending on whether chemotherapy was administered. In cases where chemotherapy was not administered, the rate of weight loss gradually decreased over time: 5.94% after a month, 3.79% after three months, and 3.01% after six months. In cases where chemotherapy was administered, the rate of weight loss fluctuated: 7.72% after a month, 8.92% after three months, and 8.10% after six months. There was a further decrease in this rate at the third month, but it was recovered by 0.82% at the sixth month. The fat-free mass loss rate of females was 5.55% (95% CI, 7.12% to 3.79%) at one month postoperative. One month after surgery, the difference in the FFM loss rates between males and females was statistically significant (*p* = 0.006). Three months after surgery, the difference in FFM loss rates between males and females was statistically significant (*p* = 0.033). Chemotherapy significantly affected (*p* = 0.042) both the time and factors in the FFM loss rates at three months after surgery. (Table 2) (Figure 1)

### 3.3. Comparison of Nutritional Intake Rates According to the Postoperative Periods by Clinical Factors 

Table 3 shows the age-adjusted effect of the clinical factors on changes in the caloric and protein intake rates after gastrectomy. The marginal mean of calorie intake was less than 30 kcal lean mass (LM) during one to three months after surgery. Calorie intake was not significant according to the clinical factors. However, protein intake was statistically significant (*p* < 0.05) over the entire postoperative period. Females showed especially significant effect interactions (*p* < 0.01) for protein intake. The intake status of micronutrient related gastric cancer for each postoperative period of the subjects is shown in Supplementary Tables S1–S3. 

### 3.4. Factors Affecting the Rate of Weight and FFM Loss 

There was a significant reduction in weight: 6.17% (95% CI, 5.61% to 6.73%) at one month after surgery, 4.89% (95% CI, 4.36% to 5.43%) at three months, and 4.17% (95% CI, 3.68% to 4.67%) at six months. The weight loss of females was significantly lower (0.63%, 95% CI, 0.14% to 1.11%) than that of males. In the chemotherapy group, the weight loss rate was significantly higher at 3.02% (95% CI, 2.46% to 3.58%) than in the group without chemotherapy. As caloric and protein intake increased, weight loss decreased, but this decrease was not significant. The fat-free mass loss rates experienced a significant reduction at one month (3.35%, 95% CI, 2.16% to 4.53%), three months (1.25%, 95% CI, 0.14% to 2.35%), and six months (1.25%, 95% CI, 0.24% to 2.26%). The FFM loss of females was significantly lower (1.94%, 95% CI, 0.20% to 3.67%) than that of males. Unlike weight loss, FFM loss showed no significant differences according to chemotherapy implementation (Table 4).

### 3.5. Factors Affecting the Weight and FFM Loss Rate According to the Postoperative Period

A multiple linear regression analysis was performed on the weight loss rates at one month, one to three months, and three to six months after surgery to determine the effects of factors affecting changes in weight and FFM loss rates. One month after surgery, women treated with chemotherapy had a significantly higher increase in their weight loss rate. One to three months postoperative, women who underwent chemotherapy still lost more weight than males. The subjects with a history of chemotherapy experienced a change in the weight loss rate that was greater than the change at one month postoperative. During this period, the intake of calories and protein did not have a significant effect on weight loss. The female FFM reduction also showed a similar tendency to weight loss status. FFM was shown to be significantly reduced in the chemotherapy group only at one to three months after surgery. Caloric intake reduction was a significant factor affecting FFM loss at one month after surgery (Table 5).

## 4. Discussion

Malnutrition is defined as a deficiency of calories, proteins, or other nutrients that can cause major changes in bodily functions [26]. Among patients with gastric cancer, malnutrition is frequent due to metabolic abnormalities, a lack of food intake, and various digestive symptoms. These are known to be major factors affecting postoperative gastric recovery and mortality [27]. While malnutrition and weight loss cannot be avoided during the period of adaptation to dietary intake after gastric cancer surgery, we can help patients recover and improve their quality of life by actively managing patients at risk of malnutrition and implementing customized nutritional interventions.

A previous study reported that 48% to 80% of patients with gastrointestinal cancer experience weight loss [28,29]. Patients with severe weight loss are more susceptible to chemotherapy’s side effects, resulting in a vicious cycle that reduces the median survival rate. Weight loss is an important independent prognostic factor for patients with cancer [30]. The average rate of postoperative weight loss in this study was 6.38% after one month, 5.06% after three months, and 4.26% after six months, displaying a recovery trend over time. The magnitude of weight loss was different between gastrectomy and chemotherapy. Weight loss was significantly lower in patients who had a proximal resection, laparoscopy, or no chemotherapy [31,32]. In our study, weight loss was affected by factors such as female gender and chemotherapy administered one to three months and three to six months after surgery. Caloric intake was a significant factor affecting weight loss one to three months after surgery and protein intake one month and three to six months after surgery. Tanabe et al. [33] performed a multiple regression analysis on the factors affecting postoperative weight loss, preoperative BMI, female sex, and surgical methods, which were independent predictors related to the degree of activities and meal intake. The mean weight loss rate was 6.1% one month after surgery and 8.3% three months after surgery, which is inconsistent with our study results. The magnitude of weight loss during chemotherapy for advanced gastrointestinal cancer patients could predict survival and has also been related to systemic inflammation [34]. Ock et al. [35] reported that a weight loss of more than 3% in the first month of chemotherapy is an independent prognostic factor for survival. In this study, the group that received chemotherapy did not show significant recovery from weight loss six at months after surgery. Muscle loss can be induced by surgery, chemotherapy, or targeted therapy and can potentially interfere with the pathways of muscle anabolism [36,37]. Patients with solid tumors and severe muscle depletion experienced more severe toxic effects when treated with adjuvant chemotherapy than patients with no apparent muscle depletion [38]. Therefore, muscle loss has provided novel targets for the treatment of cancer cachexia [36]. After gastrectomy, food intake decreases because of difficulty in ingesting a sufficient amount of food due to bloating. In addition, chemotherapy may cause various side effects, such as abdominal pain, nausea, and diarrhea, and negatively affect a patient’s nutritional status. In our study, the body fat-free mass decreased after surgery, and the factor that affected this loss was chemotherapy. Although an in-depth analysis based on the dose–response relationship has not been performed, patients undergoing chemotherapy should pay attention to their weight changes even before treatment.

The nutritional intake of patients after gastrectomy is very important. A prospective randomized controlled study [39] showed that the weight loss rate significantly decreased after a 300 kcal/day oral supplementation diet for six to eight weeks immediately after surgery. In addition, while the residual gastric capacity one year after gastrectomy was not significantly related to oral nutrition intake, increased intake can activate intestinal motility. Our study demonstrated that protein intake should be based on body composition (e.g., lean mass) rather than body weight. The variability in protein intake among all cohorts ranged from 0.83 to 1.77 g protein per kg LM per day using a theoretical protein intake of 60 g protein per day [25]. The protein intake per lean mass in all the periods after gastrectomy ranged from 1.02 to 1.28 g per day. This study analyzed the ratio of caloric and protein intake to the recommended intake after gastrectomy. Protein intake reached approximately 70% of the requirements one month postoperative, 77% at three months, and 90% at six months. The analysis of caloric intake as a factor affecting weight loss one to three months after surgery confirmed that weight loss after gastrectomy was associated with a lack of nutrient supply due to a poor diet after discharge. We investigated the level of micronutrient intake and showed a tendency for the intake of nutrients to increase over time (see Supplementary Table S1). In males, iron, vitamin A, vitamin B_6_, and vitamin C reached their recommended nutrient intake (RNI) values at six months after gastrectomy based on the Korean Dietary Reference Intakes [40], while zinc, vitamin B_1_, vitamin B_2_, niacin, folic acid, and vitamin E remained insufficient (see Supplementary Table S2). In females, vitamin A reached its RNI at one month after surgery. In addition, iron, vitamin B_6_, niacin, and vitamin C reached their RNI values at six months after surgery. However, zinc, vitamin B_1_, vitamin B_2_, folic acid, and vitamin E were still lacking (see Supplementary Table S3). In this study, various micronutrient deficiencies for each period after surgery were observed. Clinicians, therefore, must check and manage the insufficient intake of micronutrients in patients after gastrectomy.

A poor diet after gastrectomy is caused by a lack of oral intake rather than anatomical changes, thereby resulting in weight loss [4]. Hirao et al. [41] showed that active nutritional interventions improved clinical prognoses, including increased nutrient intake and shorter hospital stays. After gastrectomy, nutritional insufficiency occurs due to the unavoidable fasting period and dietary restrictions. After discharge, patients may lack sufficient vitamins and minerals due to fears regarding the consumption of some foods, such as raw vegetables, meat, and wholegrains [42]. In Korea, traditional meals, including rice, are often eaten one month after surgery. Thus, efficient and balanced meals with a high caloric density one to three months after surgery may have a more significant impact on weight loss prevention than protein-focused meals. This study has some limitations. The study period was too short to identify the factors affecting weight loss and did not include objective indicators, such as blood test results. There is a need to compare the correlation between nutritional status and muscle mass according to the various treatment conditions of cancer patients. This study, however, can be differentiated from previous studies in that it examined the dietary intake status of a large number of patients and analyzed the factors affecting weight loss by studying the changes in nutrient intake according to the observation period. Long-term research by multiple institutions is necessary to support the results of this study.

## 5. Conclusions

The factors affecting weight and FFM loss in patients undergoing gastrectomy for gastric cancer included the female sex and application chemotherapy. Caloric intake was found to be very important after surgery in preventing fat-free mass loss. The careful monitoring of weight and muscle mass changes following active nutritional interventions for sufficient nutritional support could be helpful for patients after gastrectomy. Meanwhile, results of randomized studies evaluating the benefits of nutritional monitoring and intervention during postoperative recovery are needed.

## Figures and Tables

**Table 1:** Baseline characteristics of all patients.

Variable	Total	Male	Female
	(N = 288)	(N = 165)	(N = 123)
Age, year	56.2 ± 13.1	56.9 ± 12.1	55.3 ± 14.5
Body Mass index, kg/m^2^	23.9 ± 3.5	23.5 ± 3.2	24.3 ± 3.9
Fat Mass, kg	14.2 ± 5.3	14.8 ± 6.2	13.3 ± 3.7
Fat-Free Mass, kg	49.1 ± 9.7	51.6 ± 9.5	45.7 ± 8.9
Waist circumference, cm	85.0 ± 8.0	88.2 ± 7.5	80.9 ± 6.7
Triceps skinfold thickness, mm	15.5 ± 6.5	15.6 ± 6.7	15.4 ± 6.2
Operative approach			
Laparoscopic	176 (61.1%)	107 (64.8%)	69 (56.1%)
Open	112 (38.9%)	58 (35.2%)	54 (43.9%)
Location			
Upper-third	25 (8.7%)	9 (5.5%)	16 (13.0%)
Middle-third	65 (22.6%)	37 (22.4%)	28 (22.8%)
Lower-third	198 (68.8%)	119 (72.1%)	79 (64.2%)
TNM stage			
Early gastric cancer	180 (62.5%)	107 (64.8%)	73 (59.3%)
Advanced gastric cancer	108 (37.5%)	58 (35.2%)	50 (40.7%)
Adjuvant chemotherapy			
No	217 (75.3%)	120 (72.7%)	97 (78.9%)
Yes	71 (24.7%)	45 (27.3%)	26 (21.1%)

The demographic characteristics and clinical factors of the subjects were summarized in terms of the mean and standard deviation for continuous variables and the frequency and percentage (%) for categorical variables. TNM, tumor, node, and metastasis.

**Table 2:** Changes in anthropometric values after gastrectomy according to the clinical factors.

	Variable	N	Estimated Marginal Mean * (95% CI)		
			1 Month	3 Months	6 Months
**Weight Loss Rate**	Gender ^B^				
	Male	165	−5.86 (−6.40 to −5.33)	−4.69 (−5.22 to −4.15)	−4.42 (−4.95 to −3.88)
	Female	123	-7.07 (-7.69 to -6.45)	−5.55 (−6.17 to −4.93)	−4.06 (−4.68 to −3.44)
			*p* = 0.012	*p* = 0.114	*p* = 1
	Operative approach				
	Laparoscopic	176	−6.51 (−7.04 to −5.99)	−5.33 (−5.85 to −4.81)	−4.66 (−5.18 to -4.14)
	Open	112	−6.16 (−6.82 to −5.51)	−4.63 (−5.28 to −3.98)	−3.64 (−4.29 to −2.99)
			*p* = 1	*p* = 0.309	*p* = 0.054
	TNM stage ^B^				
	Early gastric cancer	180	−6.32 (−6.84 to −5.81)	−5.02 (−5.53 to −4.50)	−3.90 (−4.42 to −3.39)
	Advanced gastric cancer	108	−6.47 (−7.13 to −5.81)	−5.12 (−5.79 to −4.46)	−4.86 (−5.53 to −4.20)
			*p* = 1	*p* = 1	*p* = 0.075
	Adjuvant chemotherapy ^A,B,C^				
	No	217	−5.94 (−6.36 to −5.52)	−3.79 (−4.21 to −3.38)	−3.01 (−3.42 to −2.59)
	Yes	71	−7.72 (−8.45 to −6.99)	−8.92 (−9.65 to −8.19)	−8.10 (−8.83 to −7.37)
			*p* < 0.001	*p* < 0.001	*p* < 0.001
**Fat-Free Mass Loss Rate**	Gender ^a^				
	Male	165	−2.18 (−3.54 to −0.81)	−0.28 (−1.64 to 1.09)	−0.83 (−2.20 to 0.53)
	Female	123	−5.55 (−7.12 to −3.97)	−2.99 (−4.57 to −1.41)	−2.07 (−3.65 to −0.49)
			***p* = 0.006**	***p* = 0.033**	*p* = 0.732
	Operative approach				
	Laparoscopic	176	−3.91 (−5.25 to −2.57)	−1.88 (−3.22 to −0.55)	−2.02 (−3.35 to −0.68)
	Open	112	−3.16 (−4.84 to −1.48)	−0.73 (−2.41 to 0.95)	−0.33 (−2.01 to 1.35)
			*p* = 1	*p* = 0.882	*p* = 0.372
	TNM stage				
	Early gastric cancer	180	−3.67 (−4.98 to −2.35)	−1.51 (−2.83 to −0.20)	−1.02 (−2.34 to 0.29)
	Advanced gastric cancer	108	−3.53 (−5.23 to −1.83)	−1.30 (−3.00 to 0.39)	−1.93 (−3.62 to −0.23)
			*p* = 1	*p* = 1	*p* = 1
	Adjuvant chemotherapy ^C^				
	No	217	−3.98 (−5.17 to −2.78)	−0.69 (1.88 to 0.50)	−0.78 (−1.97 to 0.41)
	Yes	71	−2.51 (−4.60 to −0.42)	−3.71 (−5.80 to −1.62)	−3.14 (−5.23 to −1.05)
			*p* = 0.696	*p* = 0.042	*p* = 0.165

CI, confidence interval. * Marginal means were estimated by adjusted for age from the mixed-effect model repeated measures (MMRM), with the patient as the random effect. *p*-values at each time point were corrected with Bonferroni’s correction. (^a^ *p* < 0.05 vs. factor; ^A^ *p* < 0.01 vs. factor; ^B^ *p* < 0.01 vs. time; ^C^ *p* < 0.01 vs. interaction).

**Table 3:** Change in calorie and protein intake after gastrectomy according to the clinical factors.

Variable	N	Estimated Marginal Mean * (95% CI)			
		Baseline	1 Month	3 Months	6 Months
**Calorie intake, kcal**					
Gender					
Male	165	33.59 (32.61 to 34.57)	24.69 (23.71 to 25.66)	27.43 (26.45 to 28.40)	30.20 (29.23 to 31.18)
Female	123	32.78 (31.65 to 33.92)	24.41 (23.27 to 25.54)	26.14 (25.00 to 27.27)	28.68 (27.55 to 29.81)
Operative approach					
Laparoscopic	176	33.18 (32.22 to 34.13)	24.60 (23.65 to 25.55)	27.10 (26.14 to 28.05)	29.94 (28.99 to 30.90)
Open	112	33.36 (32.16 to 34.55)	24.51 (23.31 to 25.71)	26.53 (25.33 to 27.73)	28.94 (27.74 to 30.14)
TNM stage					
Early gastric cancer	180	33.31 (32.37 to 34.25)	24.58 (23.64 to 25.52)	27.05 (26.11 to 27.99)	29.52 (28.58 to 30.46)
Advanced gastric cancer	108	33.14 (31.93 to 34.36)	24.55 (23.34 to 25.76)	26.59 (25.38 to 27.80)	29.60 (28.39 to 30.82)
Adjuvant chemotherapy					
No	217	33.24 (32.39 to 34.10)	24.49 (23.64 to 25.35)	26.82 (25.97 to 27.68)	30.02 (29.17 to 30.88)
Yes	71	33.26 (31.77 to 34.76)	24.80 (23.30 to 26.29)	27.04 (25.54 to 28.54)	28.12 (26.62 to 29.61)
**Protein intake, g**					
Gender ^B,C^					
Male	165	1.24 (1.16 to 1.31)	1.14 (1.07 to 1.21)	1.15 (1.08 to 1.22)	1.18 (1.14 to 1.26)
Female	123	1.23 (1.25 to 1.42)	1.13 (1.05 to 1.22)	1.13 (1.04 to 1.21)	1.13 (1.05 to 1.23)
Operative approach ^B^					
Laparoscopic	176	1.29 (1.22 to 1.36)	1.13 (1.06 to 1.20)	1.14 (1.05 to 1.23)	1.17 (1.10 to 1.24)
Open	112	1.25 (1.17 to 1.34)	1.15 (1.06 to 1.24)	1.15 (1.08 to 1.25)	1.14 (1.02 to 1.19)
TNM stage ^B^					
Early gastric cancer	180	1.30 (1.23 to 1.37)	1.13 (1.06 to 1.20)	1.14 (1.07 to 1.21)	1.15 (1.05 to 1.19)
Advanced gastric cancer	108	1.25 (1.16 to 1.33)	1.14 (1.05 to 1.23)	1.14 (1.06 to 1.23)	1.17 (1.10 to 1.27)
Adjuvant chemotherapy ^B^					
No	217	1.28 (1.22 to 1.35)	1.14 (1.07 to 1.20)	1.14 (1.07 to 1.20)	1.16 (1.07 to 1.28)
Yes	71	1.26 (1.15 to 1.37)	1.13 (1.02 to 1.24)	1.14 (1.03 to 1.25)	1.15 (1.04 to 1.26)

CI, confidence interval. * Marginal means were estimated by adjusted for age from the mixed-effect model repeated measures (MMRM), with the patient as the random effect. *p*-values at each time point were corrected with Bonferroni’s correction (^B^ *p* < 0.01 vs. time;^C^ *p* < 0.01 vs. interaction).

**Table 4:** Association between the clinical factors, weight, and fat-free mass (FFM) loss rate after gastrectomy.

Variable		Multivariable Model	
		Coefficient * (95% CI)	*p*-Value
**Weight Loss Rate**	F/U Time		
	Baseline	0 (Reference)	
	1 month	−6.173 (−6.732 to −5.614)	<0.001
	3 months	−4.894 (−5.425 to −4.362)	<0.001
	6 months	−4.171 (−4.666 to −3.675)	<0.001
	Age	0.002 (−0.016 to 0.021)	0.803
	Female	−0.630 (−1.111 to −0.148)	0.011
	Operative approach		
	Laparoscopic	0 (Reference)	
	Open	0.237 (−0.263 to 0.737)	0.353
	TNM stage		
	Early gastric cancer	0 (Reference)	
	Advanced gastric cancer	0.068 (−0.424 to 0.560)	0.787
	Adjuvant chemotherapy		
	No	0 (Reference)	
	Yes	−3.016 (−3.577 to −2.456)	<0.001
	Calorie intake	0.018 (−0.014 to 0.051)	0.265
	Protein intake	0.109 (−0.298 to 0.516)	0.599
**Fat-Free Mass Loss Rate**	F/U time		
	Baseline	0 (Reference)	
	1 month	−3.345 (−4.528 to −2.163)	<0.001
	3 months	−1.248 (−2.352 to −0.144)	0.027
	6 months	−1.251 (−2.259 to −0.244)	0.015
	Age	0.016 (−0.051 to 0.084)	0.630
	Female	−1.938 (−3.671 to −0.204)	0.029
	Operative approach		
	Laparoscopic	0 (Reference)	
	Open	0.988 (−0.812 to 2.788)	0.283
	TNM stage		
	Early gastric cancer	0 (Reference)	
	Advanced gastric cancer	0.118 (−1.654 to 1.891)	0.896
	Adjuvant chemotherapy		
	No	0 (Reference)	
	Yes	−0.998 (−3.018 to 1.021)	0.334
	Calorie intake	0.036 (−0.041 to 0.113)	0.355
	Protein intake	−0.107 (−0.982 to 0.768)	0.811

CI, confidence interval. * Coefficients were computed from the mixed-effect model repeated measures (MMRM), with the patient as the random effect.

**Table 5:** Fitting the multiple linear regression models for the clinical factor and weight/FFM loss rate after gastrectomy.

	Variable	Baseline to 1 Month		1 Month to 3 Months		3 Months to 6 Months	
		Coefficient * (95% CI)	*p*-Value	Coefficient * (95% CI)	*p*-Value	Coefficient * (95% CI)	*p*-Value
**Weight Loss Rate**	Age	0.017 (−0.005 to 0.038)	0.132	0.022 (−0.017 to 0.060)	0.267	−0.026 (−0.062 to 0.011)	0.166
	Female	−1.354 (−1.904 to −0.803)	<0.001	−1.213 (−2.198 to −0.228)	0.016	0.064 (−0.884 to 1.013)	0.894
	Operative approach						
	Laparoscopic	0 (Reference)		0 (Reference)		0 (Reference)	
	Open	0.211 (−0.365 to 0.787)	0.471	0.447 (−0.572 to 1.465)	0.388	0.339 (−0.642 to 1.321)	0.497
	TNM						
	Early gastric cancer	0 (Reference)		0 (Reference)		0 (Reference)	
	Advanced gastric cancer	0.117 (−0.446 to 0.680)	0.683	0.625 (−0.377 to 1.626)	0.221	−0.462 (−1.424 to 0.501)	0.346
	Adjuvant chemotherapy						
	No	0 (Reference)		0 (Reference)		0 (Reference)	
	Yes	−1.829 (−2.470 to -1.188)	<0.001	−5.179 (−6.317 to −4.042)	<0.001	−4.986 (−6.094 to −3.879)	<0.001
	Calorie intake	−0.047 (−0.114 to 0.021)	0.173	0.039 (−0.051 to 0.128)	0.395	0.046 (−0.022 to 0.113)	0.182
	Protein intake	−1.609 (−4.322 to 1.104)	0.244	1.086 (−0.793 to 2.964)	0.256	0.013 (−0.595 to 0.621)	0.966
**Fat-Free Mass Loss Rate**	Age	0.049 (−0.043 to 0.140)	0.296	0.066 (−0.030 to 0.161)	0.177	−0.024 (−0.120 to 0.072)	0.621
	Female	−3.521 (-5.867 to −1.174)	0.003	−3.288 (−5.752 to -0.824)	0.009	−1.619 (−4.118 to 0.879)	0.203
	Operative approach						
	Laparoscopic	0 (Reference)		0 (Reference)		0 (Reference)	
	Open	1.447 (−1.005 to 3.899)	0.246	1.395 (−1.151 to 3.942)	0.282	1.388 (−1.198 to 3.974)	0.292
	TNM						
	Early gastric cancer	0 (Reference)		0 (Reference)		0 (Reference)	
	Advanced gastric cancer	0.318 (−2.082 to 2.719)	0.794	0.863 (−1.641 to 3.367)	0.498	−0.616 (−3.152 to 1.919)	0.633
	Adjuvant chemotherapy						
	No	0 (Reference)		0 (Reference)		0 (Reference)	
	Yes	1.441 (−1.291 to 4.173)	0.3	−3.039 (−5.883 to −0.195)	0.036	−2.442 (−5.360 to 0.476)	0.101
	Calorie intake	−0.326 (−0.612 to −0.040)	0.026	−0.176 (−0.399 to 0.048)	0.123	0.024 (−0.153 to 0.201)	0.789
	Protein intake	8.073 (−3.485 to 19.631)	0.17	2.655 (−2.042 to 7.352)	0.267	−0.815 (−2.418 to 0.787)	0.317
	CI, confidence interval.						

CI, confidence interval. * Coefficients were computed from the mixed-effect model repeated measures (MMRM), with the patient as the random effect.

## Supplementary Materials

The following are available online at https://www.mdpi.com/2072-6643/12/7/1905/s1: Table S1: Changes in micronutrient intake per day for each postoperative period after gastrectomy (N = 288); Table S2: Changes in micronutrient intake per day for each postoperative period after gastrectomy in males (N = 165); Table S3: Changes in micronutrient intake per day for each postoperative period after gastrectomy in females.
