# Supplementary material for: Nutritional and Clinical Factors Affecting Weight and Fat-Free Mass Loss after Gastrectomy in Patients with Gastric Cancer
Source: Nutrients. 2020 Jun 27;12(7):1905. doi: 10.3390/nu12071905 (PMC7400091; doi:10.3390/nu12071905)
Supplement: Supplementary file 1 [file nutrients-12-01905-s001.pdf]

Supplementary 1. Change of micronutrient intake per day for each postoperative period after gastrectomy (N=288)

Variable	Baseline	1 month	3 months	6 months	p-value
Iron (mg)	12.42 ± 4.11	8.38 ± 3.34	8.90 ± 2.85	11.23 ± 4.32	0.014 [a, b, d]
Sodium (mg)	3,867.3 ± 1,750.3	2,911.2 ± 1,421.2	3,106.4 ± 928.6	3,937.5 ± 1,757.4	0.593
Potassium (mg)	2,439.7 ± 789.3	1,671.9 ± 674.6	1,971.9 ± 552.4	2,270.6 ± 854.8	0.005 [a]
Zinc (mg)	7.40 ± 2.75	4.99 ± 2.32	6.78 ± 2.07	6.51 ± 2.93	<0.001 [a c]
Vitamin A (mg RAE)	853.8 ± 531.0	668.2 ± 366.7	693.2 ± 214.7	782.9 ± 502.6	0.379
Vitamin B_1 (mg)	1.20 ± 0.60	0.90 ± 0.53	0.92 ± 0.22	1.01 ± 0.41	0.002 [a, b]
Vitamin B_2 (mg)	1.11 ± 0.64	0.82 ± 0.41	0.83 ± 0.44	0.92 ± 0.55	0.561
Vitamin B_6 (mg)	1.81 ± 0.73	1.20 ± 0.65	1.28 ± 0.45	1.61 ± 0.70	<0.001 [a, b, d]
Niacin (mg NE)	15.22 ± 6.90	12.9 ± 4.90	13.10 ± 4.46	14.32 ± 5.14	0.248
Vitamin C (mg)	118.32 ± 83.43	66.37 ± 60.0	82.38 ± 25.31	112.44 ± 55.10	<0.001 [a, b, d]
Folic acid (mg)	258.4 ± 124.9	180.30 ± 82.94	178.42 ± 56.50	199.97 ± 113.65	0.751
Vitamin E (mg α-TE)	11.14 ± 7.25	8.94 ± 4.72	9.87 ± 5.61	10.06 ± 6.24	0.694

The micronutrient factors of the subjects were summarized in terms of mean and standard deviation for continuous variables.

[a] there exists the significant difference between baseline and one month.

[b] there exists the significant difference between baseline and three months.

[c] there exists the significant difference between one months and three months.

[d] there exists the significant difference between one months and six months.

Supplementary 2. Change of micronutrient intake per day for each postoperative period after gastrectomy in males (N=165)

Variable	DRI	Baseline	1 month	3 months	6 months	p-value
Iron (mg)	10 [1)]	12.13 ± 3.62	8.81 ± 2.16	8.92 ± 2.87	11.25 ± 3.19	0.014 [a, b, d]
Sodium (mg)	1500 [2)]	3,928.6 ± 1,428.0	2,953.3 ± 1,456.8	3,110.2 ± 736.5	3,940.4 ± 1,468.2	0.426
Potassium (mg)	3500 [2)]	2,619.3 ± 716.8	1,692.4 ± 582.7	1,980.4 ± 590.8	2,270.6 ± 841.7	0.005 [a]
Zinc (mg)	9 [1)]	7.31 ± 1.96	5.10 ± 2.28	6.81 ± 2.10	6.78 ± 2.75	<0.001 [a c]
Vitamin A (mg RAE)	750 [1)]	861.9 ± 522.8	671.3 ± 356.4	695.6 ± 218.5	780.5 ± 481.5	0.368
Vitamin B_1 (mg)	1.2 [1)]	1.21 ± 0.45	0.91 ± 0.54	0.92 ± 0.18	1.02 ± 0.28	0.006 [a, b]
Vitamin B_2 (mg)	1.5 [1)]	1.13 ± 0.71	0.83 ± 0.36	0.84 ± 0.50	0.91 ± 0.56	0.509
Vitamin B_6 (mg)	1.5 [1)]	1.84 ± 0.59	1.21 ± 0.66	1.27 ± 0.51	1.64 ± 0.42	<0.001 [a, b, d]
Niacin (mg NE)	16 [1)]	15.49 ± 4.81	12.89 ± 5.01	13.14 ± 5.02	14.03 ± 4.69	0.446
Vitamin C (mg)	100 [1)]	119.20 ± 68.17	69.53 ± 58.7	80.51 ± 26.46	110.37 ± 60.25	0.002 [a, b, d]
Folic acid (mg)	400 [1)]	260.5 ± 120.4	185.24 ± 79.68	180.30 ± 44.28	198.56 ± 107.28	0.760
Vitamin E (mg α-TE)	12 [2)]	11.25 ± 6.83	8.95 ± 3.91	9.86 ± 5.45	10.11 ± 5.94	0.581

The micronutrient factors of the subjects were summarized in terms of mean and standard deviation for continuous variables.

DRI, Dietary Reference Intakes for Koreans 2015.

[1)] RNI, recommended nutrient intake; [2)] AI, adequate intake.

[a] there exists the significant difference between baseline and one month.

[b] there exists the significant difference between baseline and three months.

[c] there exists the significant difference between one months and three months.

[d] there exists the significant difference between one months and six months.

Supplementary 3. Change of micronutrient intake per day for each postoperative period after gastrectomy in females (N=123)

Variable	DRI	Baseline	1 month	3 months	6 months	p-value
Iron (mg)	8 [1)]	11.39 ± 5.02	7.92 ± 3.34	8.82 ± 2.79	10.95 ± 3.28	0.004 [a, d]
Sodium (mg)	1500 [2)]	3,867.3 ± 1,750.3	2,911.2 ± 1,421.2	3,101.2 ± 936.1	3,926.4 ± 1,824.6	0.272
Potassium (mg)	3500 [2)]	2,339.4 ± 716.8	1,650.3 ± 701.5	1,968.4 ± 562.0	2,271.4 ± 624.5	0.005 [a]
Zinc (mg)	7 [1)]	7.41 ± 3.15	4.68 ± 2.40	6.70 ± 3.05	6.50 ± 2.90	0.004 [a c]
Vitamin A (mg RAE)	600 [1)]	848.7 ± 628.1	680.4 ± 391.5	691.0 ± 208.6	785.9 ± 460.7	0.470
Vitamin B_1 (mg)	1.2 [1)]	1.19 ± 0.58	0.90 ± 0.53	0.94 ± 0.34	1.10 ± 0.25	0.002 [a]
Vitamin B_2 (mg)	1.2 [1)]	1.10 ± 0.71	0.84 ± 0.45	0.85 ± 0.48	0.93 ± 0.56	0.631
Vitamin B_6 (mg)	1.4 [1)]	1.79 ± 0.52	1.19 ± 0.70	1.30 ± 0.29	1.58 ± 0.43	<0.001 [a, b, d]
Niacin (mg NE)	14 [1)]	15.01 ± 5.22	12.91 ± 5.16	13.95 ± 2.68	14.31 ± 4.60	0.318
Vitamin C (mg)	100 [1)]	117.25 ± 80.19	65.24 ± 71.2	85.61 ± 24.20	113.50 ± 46.97	<0.001 [a, b]
Folic acid (mg)	400 [1)]	259.30 ± 120.8	178.61 ± 80.91	179.25 ± 63.48	200.37 ± 116.58	0.529
Vitamin E (mg α-TE)	12 [2)]	11.02 ± 5.64	8.93 ± 5.18	9.92 ± 4.82	10.03 ± 6.61	0.423

The micronutrient factors of the subjects were summarized in terms of mean and standard deviation for continuous variables.

DRI, Dietary Reference Intakes for Koreans 2015.

[1)] RNI, recommended nutrient intake; [2)] AI, adequate intake.

[a] there exists the significant difference between baseline and one month.

[b] there exists the significant difference between baseline and three months.

[c] there exists the significant difference between one months and three months.

[d] there exists the significant difference between one months and six months.
